# Association between oral health and general health indicators in older adults

**DOI:** 10.1038/s41598-018-26789-4

**Published:** 2018-06-11

**Authors:** Trung Dung Tran, Stefanie Krausch-Hofmann, Joke Duyck, Johanna de Almeida Mello, Jan De Lepeleire, Dominique Declerck, Anja Declercq, Emmanuel Lesaffre

**Affiliations:** 1 KU Leuven Biostatistics and Statistical Bioinformatics Centre (L-BioStat) - Department of Public Health and Primary Care, Leuven, Belgium; 2 KU Leuven Population Studies in Oral Health – Department of Oral Health Sciences, Leuven, Belgium; 3 KU Leuven LUCAS - Centre for Care Research and Consultancy, Leuven, Belgium; 4 KU Leuven Academic Centre for General Practice - Department of Public Health and Primary Care, Leuven, Belgium

## Abstract

Oral health (OH) and general health (GH) indicators are representations of the health status of the body. The OH indicators provide information about the oral health status while the GH indicators are used to assess the functional, cognitive, and mental conditions. OH is reported to be associated with GH. However, some specific associations, especially longitudinal relationships between OH and GH indicators, have not been fully explored. We examined the prediction ability from OH to GH and vice versa using a Belgian registry. We collected information from 8359 elderly participants, who were older than 65, lived at home, and received home care. The demographic and clinical information including three binary OH indicators and four ordinal GH indicators were collected. The participants were recorded at baseline and every six months afterwards. We opted for a generalization of a vector autoregressive model to ordinal responses. This model allows to estimate autocorrelations and cross-lagged correlations, addressing the prediction of GH from OH in a cross-sectional and longitudinal manner. We showed that individuals who had poorer OH had a higher risk of suffering from poor GH status. The percentages of correct or close prediction for GH indicators from OH indicators are high, being around 80% for all GH indicators. Additionally, having a poor OH (resp. GH) status was additionally predictive of a poor GH (resp. OH) status at following assessments. Our finding suggests using historical records of OH as well as GH indicators to draw better health care plan for geriatrics population.

## Introduction

Increasing evidence shows that OH cannot be considered as isolated from the rest of the body^1,2^. Epidemiological studies indeed indicate that OH is associated with physical, mental, and social well-being^3,4^. Kimura *et al*.^5^ shows that chewing difficulty in older adults is associated with activities of daily living, cognitive, and depression status. The association between impaired chewing function and cognition was also shown by Teixeira *et al*.^6^. Dormenval *et al*.^7^ shows an association between dryness of the mouth and poor GH for older patients. In the opposite direction, people with depression are more likely to have chewing difficulty^8^ or dryness^9^. Similarly, older adults who need help for daily activities or have cognitive impairment are more likely of having OH problems such as chewing difficulty^10,11^.

Compared to cross-sectional aspects, longitudinal aspects of the relationship between OH and GH have been studied to a lesser extent. Yamamoto *et al*.^12^ showed that OH problems predicted the development of depression. An evidence from Sweden suggested that losing teeth was associated with a decline in walking speed over time^13^. Vice versa, depression in younger ages is strongly associated with the chewing function at later ages^14^ whereas declining function ability over time is a risk of having chewing difficulties^10^. Nevertheless, some specific associations, especially longitudinal relationships between OH and GH indicators, have not been fully explored^1^.

This paper presents a longitudinal study based on the BelRAI database^15^ to explore the relationship between OH and GH by comparing the OH indicators (non-intact teeth (broken, fragmented, or loose teeth), chewing difficulty, and dry mouth) and GH indicators that summarize the functional, cognitive, and mental conditions of the subjects and their stability over time.

The first objective is to examine among older adults to what extent the GH status can be predicted from or related to the OH status. The second objective is to examine the association between the current OH and GH status with their future status. In particular, we examine the cross-lagged effects from the OH status to the GH status and vice versa.

## Methods

### Dataset

From 2010 onwards, the Belgian National Institute for Health and Disability Insurance funded initiatives to reduce the risk of institutionalization for older people, i.e keeping the older people longer at home by providing home care interventions. The elderly individuals who were at risk of institutionalization were referred to health care agencies. These agencies delivered the intervention programs at home. During the delivery of interventions, the professional caregivers, such as nurses, occupational therapists, physiotherapists, psychologists, and social workers, completed the BelRAI Home Care (HC) instrument (questionnaire), given the consent of their clients and/or family members.

BelRAI represents the Belgian version of the interRAI instruments (www.interRAI.org), which are comprehensive assessments that evaluate the physical, clinical, psychological, and social condition of people at regular intervals, in order to draw up personalized care plans^16^. From the total number of 11093 individuals in the BelRAI database, we extracted individuals older than 65 years. Subjects for whom the measurement dates were missing or for whom the date of birth was not known were excluded. Repeated assessments that were not within five to seven months of the previous assessment were also discarded. As a result, 8359 individuals were available for analysis with measurements at baseline and at every roughly six months afterwards. The number of repeated measurements varied among subjects, ranging from one to nine follow-up occasions, providing 13187 observations in total. The individuals were living at home receiving regular care. In addition, they were being assessed by caregivers working in a total of 63 home care projects. More details can be found in de Almeida Mello *et al*.^15^.

### OH and GH indicators

The OH status was evaluated via three binary OH-related items within three days before the assessment, i.e., *non-intact teeth (NT), chewing difficulty (CD)*, and *dry mouth (DM)*.

Four interRAI validated scales represent the functional, cognitive, and mental condition of the subjects and their stability and represent the GH status, i.e., Activities of Daily Living (ADL), Cognitive Performance Scale (CPS), Depression Rating Scale (DRS), and Changes in Health, End-Stage Disease, Signs, and Symptoms Scale (CHESS).

ADL is a 7-point scale (0–6) and is an indicator of functional ability of performing self-care tasks^17^. CPS concerns cognitive skills for everyday decision making, short-term memory, procedural memory, self-expression and eating^18^ and is also recorded on a 7-point scale. DRS ranges from 0 to 14 and is indicative for the mental condition of the subject with reference to symptoms of depression^19^. CHESS is a 6-point scale, which identifies subjects with unstable health conditions^20^ and points out individuals at risk of serious decline in the near future. For all scales lower values indicate better conditions or more stability.

### Other variables

Besides the OH and GH indicators, other covariates were included in the analysis: age at baseline, gender, and types of intervention (case management (59.43%), night care (16.43%), occupational therapy (10.95%), and others (9.29%)). In addition, two variables that may vary over time (time-varying covariates) were included: ‘Living status’ (alone or not), and ‘Having an informal caregivers’ (yes or no).

### Missingness information

The percentages of missingness for ADL, CPS, DRS, and having an informal caregiver varied between 5% and 7%, whereas the OH indicators had about 17% missing values. The CHESS score suffered about 23% missingness. A detailed discussion about the possible causes of missingness can be found in Vanneste *et al*.^21^. In this study we assume that the missing mechanism was missing at random^22^ (See Supplementary Note S1). This implies that the probability of being missing for a particular observation can depend on anything but not the missing value itself.

### Statistical analysis

Two analyses were conducted in order to examine the relationship between OH and GH indicators. First, each GH indicator was regressed on the OH indicators using a random effects proportional odds model^23^ with age at baseline, gender, and types of intervention, time, the interactions between types of intervention and time, living status, and having an informal caregiver as covariates (See Supplementary Note S2). Missing values are imputed by making use of a sequence of Bayesian imputation models (See Supplementary Note S3).

For each GH indicator, the contingency table of the observed and predicted values is constructed and the percentage of concordance was calculated. A higher value of this percentage means that the model fits better the observed data.

We examined in the second analysis whether the current OH status can give additional information for the future GH status given the current GH status and vice versa. To this end, we fitted a bivariate ordinal autoregressive model with the OH and GH indicators at a particular examination as responses and their values at the previous examination as covariates (See Supplementary Fig. S1 for the hypothesized relationship between oral health (OH) and general health (OH) over time and Supplementary Note S4 for more information about model specification).

All the analyses were performed in Bayesian framework with vague priors^24^. The models were implemented in R (version 3.2.5, https://www.r-project.org/) using runjags (version 2.0.4-2, http://mcmc-jags.sourceforge.net/) and rstan packages (version 2.16, http://mc-stan.org/). Four chains were run for each analysis. Convergence was checked by examining the trace plots and using Brooks–Gelman–Rubin diagnostic. Finally the converged chains were run until the Monte Carlo standard error was less than 5% of the posterior standard deviation^24^.

### Data availability

The data used in this study is under the permission from the Belgian Privacy Commission. However the authority body does not allow us to give the raw data to a third party. The same request can be made by any researcher and the permission can be given. For access to the database, the Belgian Privacy Commission should be contacted at commission@privacycommission.be (www.privacycommission.be).

### Ethical considerations

The Ethics committee of two Belgian universities (Université Catholique de Louvain and KU Leuven (B40320108337)) and the Belgian Privacy Commission jointly approved the study. A formal procedure was implemented in order to make sure the privacy of the data. The participants were asked to sign an informed consent. In cases of not capable of signing the consent, a legal representative by the Belgian law signed it on their behalf. Participation can be withdrawn at any time, without any consequences for the care they received. All data were anonymized before being used by a third party.

## Results

The population analyzed in the study consisted of 8359 older people with mean age of 81.2 (SD = 7.0, range 65–102) and 68.3% were female. About 55% of older people lived alone and 78.6% had an informal caregiver.

### First analysis

The results for the association between OH and GH indicators of the first analysis are provided in Table 1. Among the three OH indicators, chewing difficulty was most related to GH status as it showed highly associated with all GH indicators. Non-intact teeth was also highly associated with CPS, DRS, and CHESS whereas dry mouth was highly associated with ADL, DRS, and CHESS.

**Table 1:** Odds ratios (OR) of being poor general health status (ADL, CPS, DRS, and CHESS) and 95% credible intervals (CI) for individuals having non-intact teeth (NT), chewing difficulty (CD), and dry mouth (DM) compared to individuals without these oral health problems.

Effect	OR	95% CI	
**ADL**			
NT	1.195	0.920	1.556
CD	3.452	2.632	4.550
DM	1.390	1.087	1.800
**CPS**			
NT	2.378	1.628	3.464
CD	10.886	7.348	16.287
DM	0.967	0.682	1.366
**DRS**			
NT	1.709	1.299	2.257
CD	3.729	2.769	5.040
DM	3.711	2.845	4.860
**CHESS**			
NT	1.287	1.070	1.549
CD	3.102	2.524	3.825
DM	2.686	2.228	3.232

ADL: Activities of Daily Living.

CPS: Cognitive Performance Scale.

DRS: Depression Rating Scale.

CHESS: Changes in Health, End-Stage Disease, Signs, and Symptoms Scale.

It was also noticed that almost all estimated odds ratios were greater than one, which means that compared to people who do not have OH problems, individuals who show poorer OH present a higher risk of suffering from poor GH status. The odds ratios and 95% credible intervals for the other covariates are given in Supplementary Tables S1, S2, S3 and S4.

As reported in Table 2, around 81.65% of the ADL observations were predicted correctly and around 96.89% predicted within one-unit of the observed value. For the other GH indicators, the percentages for CPS were 91.09% and 98.67% respectively, for DRS 73.97% and 93.03% respectively, and CHESS 73.98% and 97.54% respectively. The contingency tables for these GH indicators are provided in Supplementary Tables S5, S6 and S7.

**Table 2:** Contingency table of the observed and fitted values along with row-wise percentages for ADL.

Observed value	Predicted values^*^						
	0	1	2	3	4	5	6
0	2939	282	167	28	0	1	0
	86.0^**^	8.3	4.9	0.8	0.0	0.0	0.0
1	140	571	312	31	0	0	0
	13.3	54.2	29.6	2.9	0.0	0.0	0.0
2	31	243	1801	144	2	0	0
	1.4	10.9	81.1	6.5	0.1	0.0	0.0
3	5	14	206	2789	50	2	0
	0.2	0.5	6.7	91.0	1.6	0.1	0.0
4	2	3	31	204	1168	22	0
	0.1	0.2	2.2	14.3	81.7	1.5	0.0
5	0	2	12	46	183	677	0
	0.0	0.2	1.3	5.0	19.9	73.6	0.0
6	0	0	0	3	2	93	118
	0.0	0.0	0.0	1.4	0.9	43.1	54.6

*Predicted values are taken as the median of the corresponding posterior sample.

**The row-wise percentages.

### Second analysis

**Figure 1:**
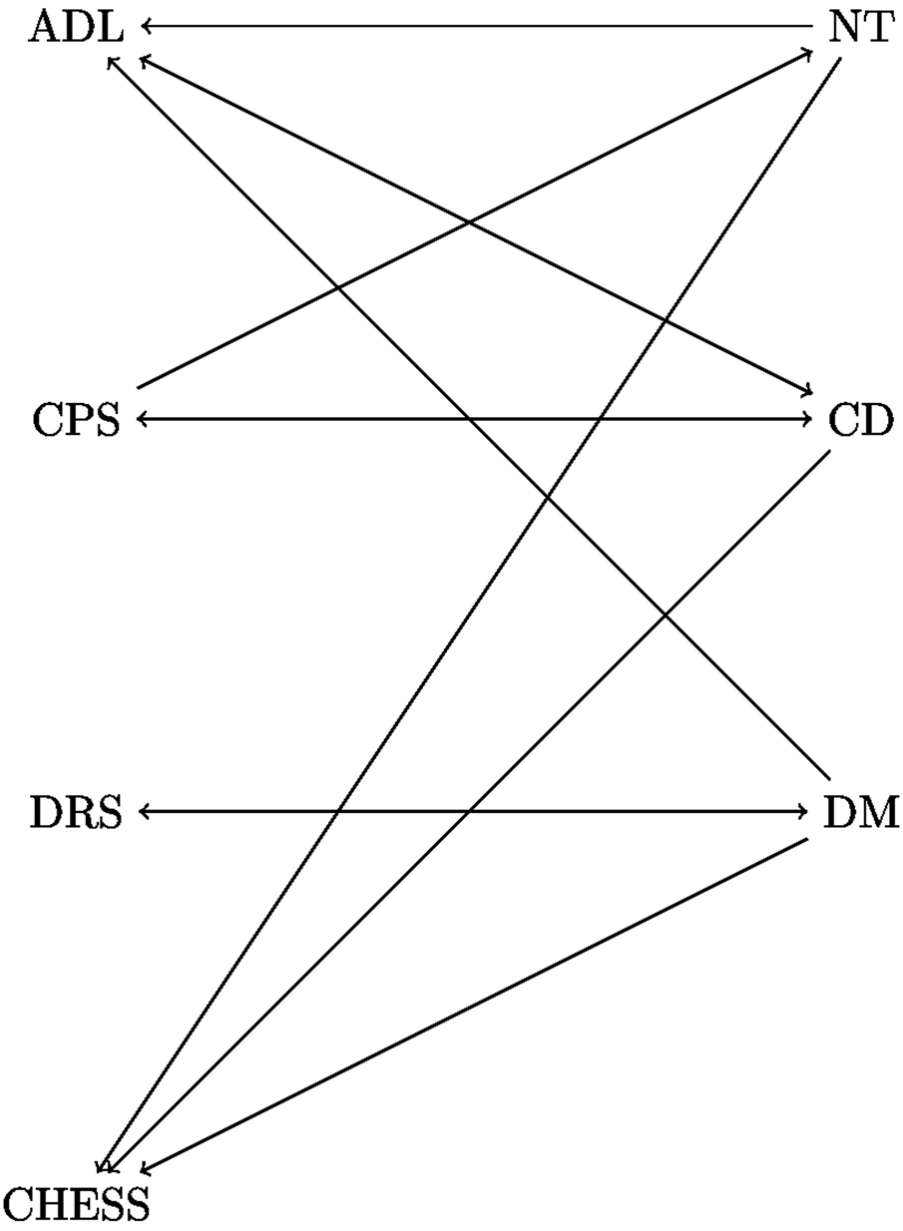
Significant associations of the current OH (resp. GH) status to the future GH (resp. OH) status, represented by OH and GH indicators. The indicators are non-intact teeth (NT), chewing difficulty (CD), dry mouth (DM), Activities of Daily Living (ADL), Cognitive Performance Scale (CPS), Depression Rating Scale (DRS), and Changes in Health, End-Stage Disease, Signs, and Symptoms Scale (CHESS). The arrows indicate the direction of association. For example the arrow from CD to ADL means that CD is additionally predictive of ADL in the future.

Secondly, twelve bivariate autoregressive models for the pairs between one OH and one GH indicator were fitted. The second analysis gives extra information about the association between OH and GH indicators, i.e. we examined OH and GH at a time point and related their values to the corresponding ones a previous time point. The estimate for the cross-lagged parameters (i.e. *γ*_12_ (*resp γ*_21_)) (95% CI) for the pair of ADL and chewing difficulty was 0.077 (0.043, 0.111) (resp. 0.031 (0.003, 0.059)). This indicates that the current chewing difficulty (resp. ADL) provides a significant amount of information in predicting the future value of ADL (resp. chewing difficulty). In addition, higher values for current chewing difficulty corresponds to higher values of future ADL. In other words, having a chewing problem is additionally predictive of a poor ADL status at the next examination. Similarly we also see that current ADL has extra predictive ability for chewing difficulty at the next assessment. The results for all the pairs are depicted in Fig. 1 where significant effects are shown with the arrows pointing to the direction of the prediction, e.g. the arrow from CD to CHESS means that given current CHESS, chewing difficulty is still predictive of CHESS in the future. Parameter estimates for the other pairs are given in Supplementary Table S8.

## Discussion

Since OH is reported to be associated with general health^25^, it is important to evaluate and investigate this association and to gain insight the underlying mechanisms. For this reason better understanding of the cross-sectional and longitudinal relationship between OH and GH is necessary. Using the BelRAI database we have shown that (i) OH and GH indicators are highly correlated and (ii) given the current GH (resp. OH) status, the current OH (resp. GH) status is additionally predictable of the future GH (resp. OH) status.

The important associations between OH and GH indicators using longitudinal data are consistent with findings from previous cross-sectional and longitudinal studies^5–12^. For example, we confirm the finding in Japan^26^ that GH is associated with a change in chewing ability after three years. Similar to the conclusion on tooth loss^13^, we agree that having non-intact teeth is predictive of poor ADL in the future. Nevertheless, we further provide information on how accurate it is to make a prediction for GH indicators based on OH indicators. Moreover, we examine the cross-lagged effects given the autoregressive effects. These findings of the important cross-lagged effects cannot be compared to other studies because of differences in study designs.

The observed associations between OH and GH may have clinical implications. In making predictions for future GH indicators, our study suggests to include OH indicators in the past as predictors and vice versa. Because of the cross-lagged effects, this study can provide empirical evidence for studying possible pathways from OH to GH indicators and vice versa^27–30^. In addition, it can server as an exploratory tool for further analysis on causal relationship between OH and GH. The findings also suggest to take care of mobility, cognition, and depression problems in order to reduce the risk for poor oral health.

A limitation of the present study is that there is a portion of individuals having only one measurement. In addition, the average number of repeated measurements for those who have at least two assessments is 2.4 (measurements), i.e. about 9 months. This might hinder detecting effects that need more repeated measurements per subject or need longer time to emerge.

## Conclusions

A careful assessment of disability and impairment of OH should be included in geriatric assessments in order to define the health care plan. The importance of the cross-lagged effects suggests that using the historical records of OH as well as GH indicators can help practitioners and dentists draw better health care plan for geriatrics population.

## Electronic supplementary material

Supplementary information
