# Supplementary material for: Association between oral health and general health indicators in older adults
Source: Sci Rep. 2018 Jun 11;8:8871. doi: 10.1038/s41598-018-26789-4 (PMC5996062; doi:10.1038/s41598-018-26789-4)
Supplement: Supplementary file 1 — Supplementary information [file 41598_2018_26789_MOESM1_ESM.pdf]

Supplementary information of the manuscript

"**Association between oral health and general health indicators in older adults**"

Trung Dung Tran, MSc[1], Stefanie Krausch-Hofmann, MSc[2], Joke Duyck, PhD[2], Johanna de Almeida Mello, Ms Econ[3], Jan De Lepeleire, MD, PhD[4], Dominique Declerck, PhD[2], Anja Declercq, PhD[3], Emmanuel Lesaffre, Dr. Sc.[1]

[1] KU Leuven Biostatistics and Statistical Bioinformatics Centre (L-BioStat) - Department of Public Health and Primary Care

[2] KU Leuven Population Studies in Oral Health – Department of Oral Health Sciences

[3] KU Leuven LUCAS - Centre for Care Research and Consultancy

[4] KU Leuven Academic Centre for General Practice - Department of Public Health and Primary Care

The corresponding author: Trung Dung Tran, Kapucijnenvoer 35 blok d - bus 7001
3000 Leuven, Belgium, tel. +32 16 32 26 96, e-mail: trungdung.tran@kuleuven.be

Supplementary Note S1. Missing mechanism.

Missing covariates were imputed via a sequence of imputation models, avoiding deleting missing rows as default option in the standard software packages such as SAS. This procedure is valid, yielding unbiased estimates when the missing mechanism is missing at random, i.e. the probability of being missing depends on the set of observed values but is unrelated to the specific missing values that would have been obtained. However, this assumption might be violated when, for example, individuals do not provide information about their mouth because of hesitating to show their problem. This assumption cannot be tested empirically by the data at hand.

Supplementary Note S2. Model specification for the random effects proportional odds model.

$$logit\left(P(GH_{ij} \le m)\right)$$
$$= \alpha_m$$
$$+ \beta_1 age_i + \beta_2 gender_i + \beta_3 CM_i + \beta_4 NC_i + \beta_5 OT_i$$
$$+ (\beta_6 + \beta_7 CM_i + \beta_8 NC_i + \beta_9 OT_i) year_j + \beta_{10} alone_{ij} + \beta_{11} ifc_{ij}$$
$$+ \beta_{12} NT_{ij} + \beta_{13} CD_{ij} + \beta_{14} DM_{ij} + u_i.$$

where $P(GH_{ij} \le m)$ ($i = 1, \dots, 8359$, $j = 1, \dots, T_i$ with T_i is the number of repeated measurements for the ith individual) is the probability that the GH indicator of the ith individual at the jth occasion is less than or equal to m with $m_{min} \le m < m_{max}$, and m_{min}, m_{max} are the minimum and maximum scores respectively of the GH indicators, and $u_i \sim N(0, \sigma_u^2)$.

The model includes time-independent covariates (age at baseline, gender, and types of intervention), time, the interactions between types of intervention and time, and time-varying covariates (living status and having an informal caregiver). (See Supplementary Table S9 for the values of the covariates in the model).

Supplementary Note S3. Multiple imputation in Bayesian fitting framework that was implemented for fitting the random effects proportional odds model.

At each iteration, the first variable with missing values, ifc, was regressed on all the fully observed covariates using a random intercept logistic regression model. From this regression model, ifc was imputed and the imputed value will be available for any model that use ifc as a covariate. In the next imputation model, NT was imputed using a random intercept logistic regression with all the fully observed covariates and ifc. We do the same for CD. Finally DM was imputed by regressing on the fully observed covariates and previous imputed

covariates. After this procedure all covariates on the right-hand side of the random effects proportional odds model were available for estimating the regression parameters and other parameters of that model.

Supplementary Note S4. Model specification for the bivariate autoregressive model.

To do that, we first assumed that each ordinal/binary variable was a manifestation of an underlying standard normal one, discretized by a set of cut-points. That means for a GH indicator with $c + 1$ categories, denotes GH^* the underlying standard normally distributed variable, i.e. $GH^* \sim N(0, 1)$, and $\alpha = (\alpha_0, \alpha_{1,} \ldots, \alpha_{c-1})^T$ the vector of c cut-points then:

$$GH = k \quad if\ and\ only\ if \quad \alpha_{k-1} < GH^* \leq \alpha_k,$$

where $\alpha_{-1} = -\infty, \alpha_c = +\infty$. Analogous definition was applied for each OH indicator and we denoted the underlying variable as OH^*.

Using underlying continuous variables, the following linear regression models were fitted simultaneously:

$$GH_t^* = \gamma_{11} GH_{t-1}^* + \gamma_{12} OH_{t-1}^* + \delta_1,$$

$$OH_t^* = \gamma_{22} OH_{t-1}^* + \gamma_{21} GH_{t-1}^* + \delta_2,$$

where δ_1 and δ_2 were assumed to follow a normal distribution with mean 0 and variances $1 - {\gamma_{11}}^2 - {\gamma_{12}}^2$ and $1 - {\gamma_{22}}^2 - {\gamma_{21}}^2$ respectively. Through the latent variables, the models assume that both OH and GH status at a particular time point jointly explain the OH and GH status in the future and these associations can be described by the two above linear functions. Parameter γ_{11} $(resp. \gamma_{22})$ represents the fact that the current status of GH (resp. OH) provides information for the future status of GH (resp. OH). In addition to that, the cross-lagged parameter γ_{12} $(resp. \gamma_{21})$ indicates the amount of extra information that the current OH (resp. GH) indicator provides the future GH (resp. OH) indicator.

Reference: Lutkepohl, H. Introduction to Multiple Time Series Analysis. Berlin: Springer-Verlag, 1993.

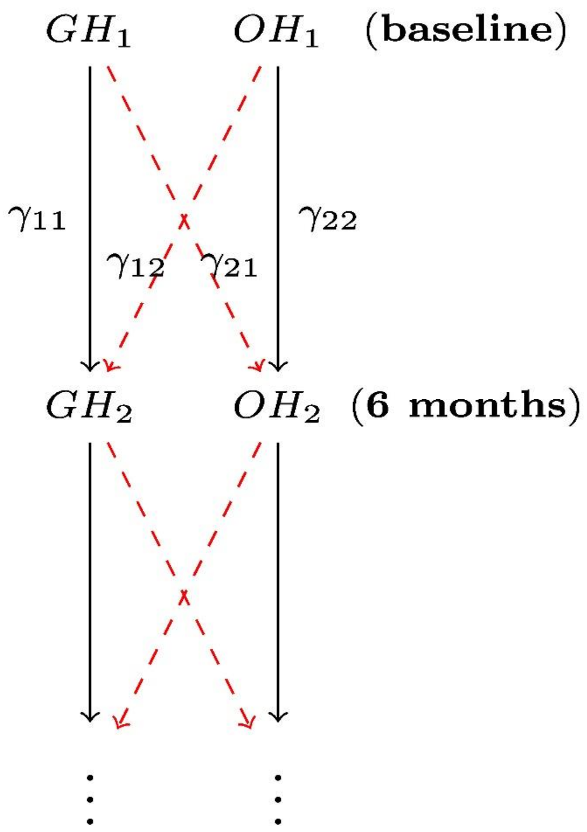

Supplementary Figure S1. Hypothesized relationship between oral health (OH) and general health (OH) over time. The autoregressive (γ_{11}) and cross-lagged (γ_{12}) parameters represent the effect of GH and OH respectively on GH in the future. Similarly the autoregressive (γ_{22}) and cross-lagged (γ_{21}) parameters represent the effect of OH and GH respectively on OH in the future.

Supplementary Table S1. Odds ratios (OR) of being poor ADL and 95% credible intervals (CI) for all effects in the first analysis.

Effect	OR	95% CI	
age	1.451	1.316	1.601
gender	0.885	0.716	1.089
CM	1.904	1.410	2.569
NC	13.561	9.430	19.555
OT	2.650	1.803	3.956
year	1.197	0.870	1.641
CM *year	0.522	0.373	0.738
NC * year	0.925	0.620	1.403
OT * year	1.126	0.618	2.058
alone	0.201	0.165	0.243
ex	2.656	2.133	3.328
NT	1.195	0.920	1.556
CD	3.452	2.632	4.550
DM	1.390	1.087	1.800

CM: case management
NC: night care
OT: occupational therapy
ifc: Having informal caregiver
NT: Non-intact teeth
CD: Chewing difficulty
DM: Dry mouth

Supplementary Table S2. Odds ratios (OR) of being poor CPS and 95% credible intervals (CI) for all effects in the first analysis.

Effect	OR	95% CI	
age	1.692	1.451	1.967
gender	0.355	0.256	0.485
CM	0.067	0.043	0.104
NC	1.963	1.163	3.346
OT	0.174	0.095	0.317
year	1.645	1.182	2.293
CM *year	1.478	1.028	2.122
NC * year	1.131	0.727	1.745
OT * year	1.476	0.711	3.020
alone	0.083	0.063	0.110
ex	4.787	3.547	6.450
NT	2.378	1.628	3.464
CD	10.886	7.348	16.287
DM	0.967	0.682	1.366

CM: case management
NC: night care
OT: occupational therapy
ifc: Having informal caregiver
NT: Non-intact teeth
CD: Chewing difficulty
DM: Dry mouth

Supplementary Table S3. Odds ratios (OR) of being poor DRS and 95% credible intervals (CI) for all effects in the first analysis.

Effect	OR	95% CI	
age	0.752	0.676	0.837
gender	1.267	1.000	1.604
CM	0.105	0.076	0.146
NC	0.509	0.349	0.750
OT	0.127	0.082	0.201
year	0.845	0.644	1.091
CM *year	1.333	0.999	1.816
NC * year	2.113	1.449	3.133
OT * year	1.002	0.536	1.876
alone	0.474	0.387	0.582
ex	0.635	0.506	0.807
NT	1.709	1.299	2.257
CD	3.729	2.769	5.040
DM	3.711	2.845	4.860

CM: case management
NC: night care
OT: occupational therapy
ifc: Having informal caregiver
NT: Non-intact teeth
CD: Chewing difficulty
DM: Dry mouth

Supplementary Table S4. Odds ratios (OR) of being poor CHESS and 95% credible intervals (CI) for all effects in the first analysis

Effect	OR	95% CI	
age	1.099	1.028	1.177
gender	0.765	0.660	0.888
CM	2.021	1.645	2.492
NC	2.940	2.264	3.856
OT	2.711	2.031	3.622
year	0.778	0.573	1.051
CM *year	0.512	0.365	0.718
NC * year	0.616	0.399	0.951
OT * year	0.342	0.177	0.664
alone	0.843	0.735	0.964
ex	1.692	1.429	2.012
NT	1.287	1.070	1.549
CD	3.102	2.524	3.825
DM	2.686	2.228	3.232

CM: case management
NC: night care
OT: occupational therapy
ifc: Having informal caregiver
NT: Non-intact teeth
CD: Chewing difficulty
DM: Dry mouth

Supplementary Table S5. Contingency table of the observed and fitted values for CPS.

	Predicted value*						
Observed value	0	1	2	3	4	5	6
0	4801	155	25	2	2	0	0
	96.3**	3.1	0.5	0.0	0.0	0.0	0.0
1	104	1744	135	25	0	1	0
	5.2	86.8	6.7	1.2	0.0	0.0	0.0
2	10	117	1543	75	5	0	0
	0.6	6.7	88.2	4.3	0.3	0.0	0.0
3	3	31	129	1501	41	1	0
	0.2	1.8	7.6	88.0	2.4	0.1	0.0
4	0	2	13	103	785	19	1
	0.0	0.2	1.4	11.2	85.0	2.1	0.1
5	0	1	10	29	51	787	2
	0.0	0.1	1.1	3.3	5.8	89.4	0.2
6	0	0	2	1	1	10	146
	0.0	0.0	1.3	0.6	0.6	6.3	91.3

* Predicted values are taken as the median of the corresponding posterior sample.
** The row-wise percentages

Supplementary Table S6. Contingency table of the observed and fitted values for DRS.

	Predicted value*														
Observed value	0	1	2	3	4	5	6	7	8	9	10	11	12	13	14
0	5715	203	56	15	6	0	2	0	0	0	0	0	0	0	0
	95.3**	3.39	0.93	0.3	0.1	0	0	0	0	0	0	0	0	0	0
1	180	1175	128	27	6	4	0	0	0	0	0	0	0	0	0
	11.8	77.3	8.42	1.8	0.4	0.3	0	0	0	0	0	0	0	0	0
2	32	166	1067	96	31	1	1	2	0	0	0	0	0	0	0
	2.29	11.9	76.4	6.9	2.2	0.1	0.1	0	0	0	0	0	0	0	0
3	9	63	251	551	64	13	4	0	1	0	0	0	0	0	0
	0.94	6.59	26.3	58	6.7	1.4	0.4	0	0	0	0	0	0	0	0
4	6	19	54	327	384	33	12	1	1	0	0	0	0	0	0
	0.72	2.27	6.45	39	46	3.9	1.4	0	0	0	0	0	0	0	0
5	2	2	9	28	287	102	23	8	1	0	0	0	0	0	0
	0.43	0.43	1.95	6.1	62	22	5	2	0	0	0	0	0	0	0
6	2	3	6	21	42	255	82	8	3	1	0	0	0	0	0
	0.47	0.71	1.42	5	9.9	60	19	2	1	0	0	0	0	0	0
7	2	0	11	7	15	25	146	47	10	2	1	0	0	0	0
	0.75	0	4.14	2.6	5.6	9.4	55	18	4	1	0	0	0	0	0
8	0	1	3	10	5	12	43	97	20	2	3	0	0	0	0
	0	0.51	1.53	5.1	2.6	6.1	22	49	10	1	2	0	0	0	0
9	0	0	1	2	4	2	7	44	44	12	4	1	0	0	0
	0	0	0.83	1.7	3.3	1.7	5.8	36	36	10	3	1	0	0	0
10	0	0	2	1	1	0	3	9	44	13	5	1	1	0	0
	0	0	2.5	1.3	1.3	0	3.8	11	55	16	6	1	1	0	0
11	0	0	3	2	3	1	4	0	7	17	5	4	0	0	0
	0	0	6.52	4.3	6.5	2.2	8.7	0	15	37	11	9	0	0	0
12	0	0	0	1	1	0	3	1	4	15	16	7	0	0	0
	0	0	0	2.1	2.1	0	6.3	2	8	31	33	15	0	0	0
13	0	0	1	0	0	0	0	1	1	0	6	5	7	2	0
	0	0	4.35	0	0	0	0	4	4	0	26	22	30	9	0
14	0	1	1	0	0	0	0	0	1	2	1	3	8	7	10
	0	2.94	2.94	0	0	0	0	0	3	6	3	9	24	21	29

* Predicted values are taken as the median of the corresponding posterior sample.
** The row-wise percentages

Supplementary Table S7. Contingency table of the observed and fitted values for CHESS.

	Predicted value*				
Observed value	0	1	2	3	4
0	3220	538	11	0	0
	85.4**	14.3	0.3	0.0	0.0
1	177	3236	75	3	0
	5.1	92.7	2.1	0.1	0.0
2	17	879	966	8	0
	0.9	47.0	51.7	0.4	0.0
3	4	59	566	52	1
	0.6	8.7	83.0	7.6	0.1
4	0	9	105	121	5
	0.0	3.8	43.8	50.4	2.1
5	1	1	2	36	17
	1.8	1.8	3.5	63.2	29.8

* Predicted values are taken as the median of the corresponding posterior sample.
** The row-wise percentages

Supplementary Table S8. Parameter estimates and 95% credible intervals (CI) for all $\gamma's$ for all pairs of one OH and one GH indicator.

	γ_{11}			γ_{12}			γ_{21}			γ_{22}		
Pair	Est.	95% CI		Est.	95% CI		Est.	95% CI		Est.	95% CI	
ADL and NT	0.83	0.82	0.84	0.03	0.00	0.06	0.00	-0.02	0.03	0.98	0.98	0.99
ADL and CD	0.83	0.82	0.84	0.08	0.04	0.11	0.03	0.00	0.06	0.97	0.95	0.98
ADL and DM	0.83	0.82	0.84	0.07	0.04	0.11	0.00	-0.03	0.03	0.96	0.95	0.97
CPS and NT	0.93	0.93	0.94	0.02	0.00	0.04	0.03	0.01	0.06	0.98	0.98	0.99
CPS and CD	0.93	0.93	0.94	0.03	0.01	0.05	0.05	0.02	0.08	0.96	0.95	0.97
CPS and DM	0.93	0.93	0.94	0.02	-0.01	0.04	0.02	-0.01	0.06	0.96	0.95	0.97
DRS and NT	0.88	0.86	0.89	0.01	-0.02	0.03	0.01	-0.02	0.03	0.98	0.98	0.99
DRS and CD	0.87	0.86	0.88	0.02	-0.01	0.05	0.01	-0.02	0.04	0.97	0.95	0.98
DRS and DM	0.87	0.86	0.88	0.04	0.01	0.07	0.05	0.01	0.08	0.96	0.94	0.97
CHESS and NT	0.57	0.54	0.60	0.09	0.05	0.14	0.02	-0.01	0.04	0.98	0.98	0.99
CHESS and CD	0.56	0.53	0.59	0.18	0.13	0.23	0.01	-0.02	0.05	0.97	0.96	0.98
CHESS and DM	0.55	0.52	0.59	0.23	0.18	0.28	0.00	-0.03	0.03	0.96	0.95	0.97

Supplementary Table S9. Covariates for the first analysis.

Notation	Covariate	Values
age	Age at baseline, after standardized by mean=81.2, sd=0.7	$age =$ (Age at baseline – 81.2)/0.7
$gender$	Gender	1 female, 0 male
CM<br><br>NC<br>OT	Dummy variables for type of interventions	CM: 1 if the intervention is case management, 0 otherwise<br>NC: 1 if night care, 0 otherwise<br>TO: 1 if occupational therapy, 0 otherwise
$year$	Time of measurement	0, 0.5, 1, 1.5, 2, 2.5, 3, 3.5, 4
$alone$	Living status	1 if live alone, 0 otherwise
ifc	Having informal caregiver	1 if having an informal caregiver, 0 otherwise
NT	Non-intact teeth	1 if having non-intact teeth, 0 otherwise
CD	Chewing difficulty	1 if having chewing difficulty, 0 otherwise
DM	Dry mouth	1 if having dry mouth, 0 otherwise
